# Supplementary material for: Ursodeoxycholic acid reduces antitumor immunosuppression by inducing CHIP-mediated TGF-β degradation
Source: Nat Commun. 2022 Jun 14;13:3419. doi: 10.1038/s41467-022-31141-6 (PMC9198048; doi:10.1038/s41467-022-31141-6)
Supplement: Supplementary file 1 — Supplementary Information [file 41467_2022_31141_MOESM1_ESM.pdf]

Supplementary information

Ursodeoxycholic acid reduces antitumor immunosuppression by inducing CHIP-mediated TGF-β degradation

Shen et al.

Supplementary figures 1 to 11

Supplementary tables 1 to 4

Shen et al.

Supplementary Figure 1

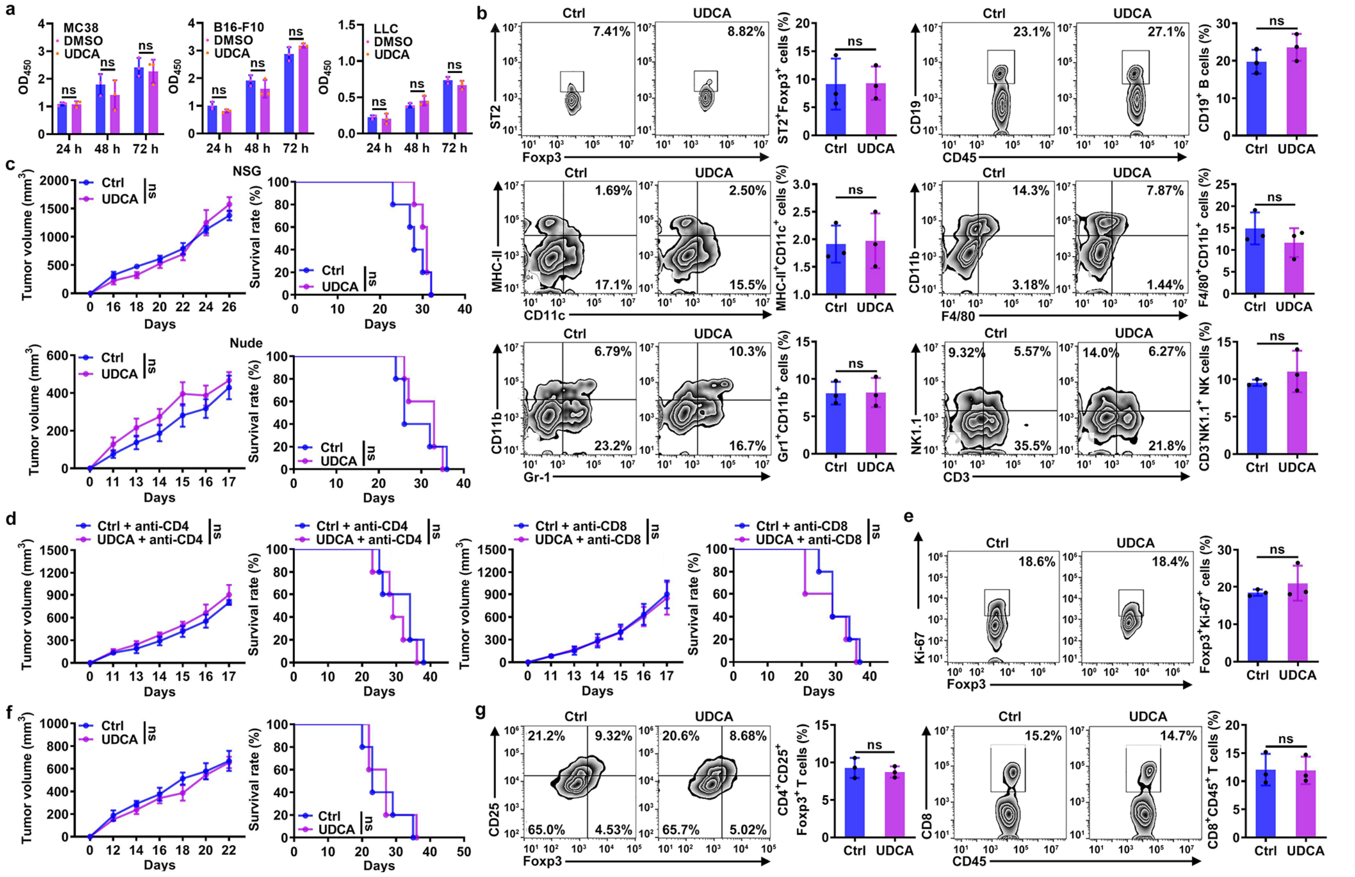

Supplementary Figure 1 UDCA inhibits Treg cell differentiation in tumor mice. **a** CCK-8 analysis of the viability of MC38, B16-F10, and LLC cells treated with 50 μM UDCA for the indicated time. **b** FC analysis of the indicated cell subsets in CD45$^+$ TILs from LLC tumor-bearing mice that received i.p. injection of 30 mg kg^{-1} UDCA every 2 days on day 18. **c** Tumor sizes and mouse survival of LLC tumor-bearing NSG and nude mice that received i.p. injection with 30 mg kg^{-1} UDCA every 2 days. **d** Tumor sizes and mouse survival of LLC tumor-bearing mice that received i.p. injection of 30 mg kg^{-1} UDCA every 2 days and 120 μg anti-CD4-neutralizing antibody (anti-CD4) or 40 μg anti-CD8 every 3 days. **e** FC analysis of Ki-67$^+$ Treg cells in TILs of LLC tumor-bearing mice that received i.p. injection of 30 mg kg^{-1} UDCA every 2 days on day 18. **f**, **g** Tumor sizes and mouse survival (**f**) and FC analysis of CD4$^+$CD25$^+$Foxp3$^+$ Treg cells and CD8$^+$CD45$^+$ T cells in TILs on day 18 (**g**) of LLC tumor-bearing *Smad3*$^{-/-}$ mice that received i.p. injection with 30 mg kg^{-1} UDCA every 2 days. Representative results from

two (**a**) or three (**b-g**) independent experiments are shown (*n* = 3 in **a**, **b**, **e**, **g**; *n* = 5 in **c**, **d**, **f**). ns, not significant (unpaired two-tailed Student's *t*-test except log-rank test was used for survival rate analysis; mean and s.d.).

Supplementary Figure 2

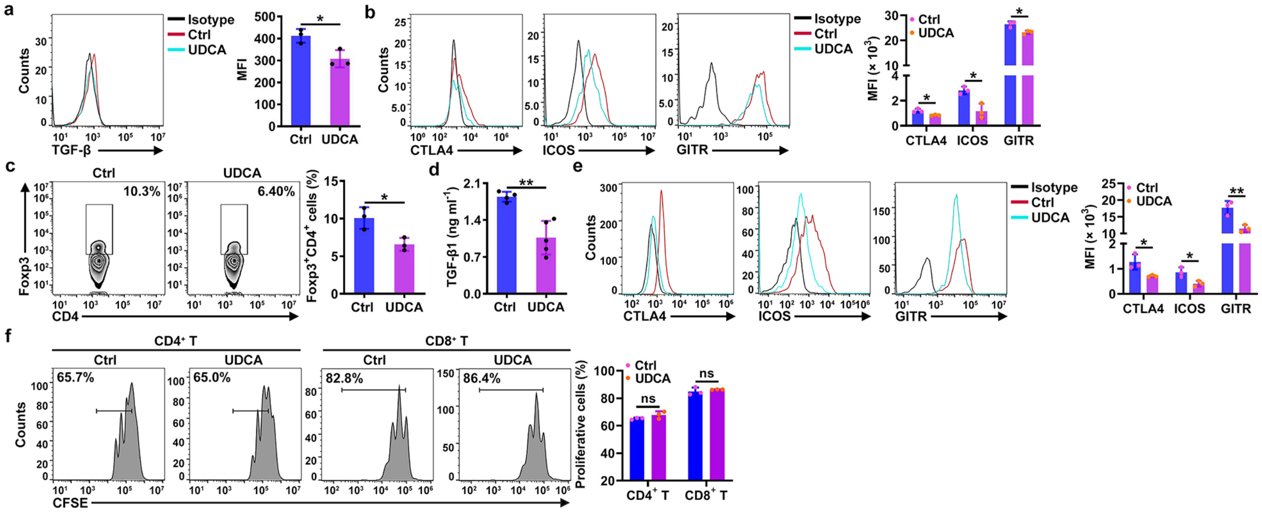

Supplementary Figure 2 UDCA inhibits Treg cell activation in tumor mice. **a-e** FC analysis of TGF-β (**a**), CTLA4, ICOS and GITR (**b**) on Treg cells in TILs, Treg cells in the spleen (**c**) and ELISA analysis of TGF-β1 protein in the supernatants of splenic $CD4^+CD25^+$ Treg cells stimulated with anti-CD3 and anti-CD28 for 48 h (**d**) and FC analysis of CTLA4, ICOS and GITR on Treg cells in the spleen (**e**) of LLC tumor-bearing mice received i.p. injection of 30 mg kg^{-1} UDCA every 2 days on day 18. **f** FC analysis of the proliferation of CFSE-labeled $CD4^+$ or $CD8^+$ T cells cocultured with $CD4^+CD25^+$ Treg cells isolated from the spleen of LLC tumor-bearing *Tgfbr2*$^{f/f}$*Er-cre* mice that received i.p. injection of 30 mg kg^{-1} UDCA every 2 days on day 18 at a 4:1 ratio in anti-CD3 and anti-CD28-coated plates for 5 days. Representative results from three independent experiments are shown (n = 3 in **a-c**, **e**, **f**; n = 4 in **d**). *P < 0.05; **P < 0.01 and ns, not significant (unpaired two-tailed Student's t-test; mean and s.d.). See Source Data file for the exact P-values.

Supplementary Figure 3

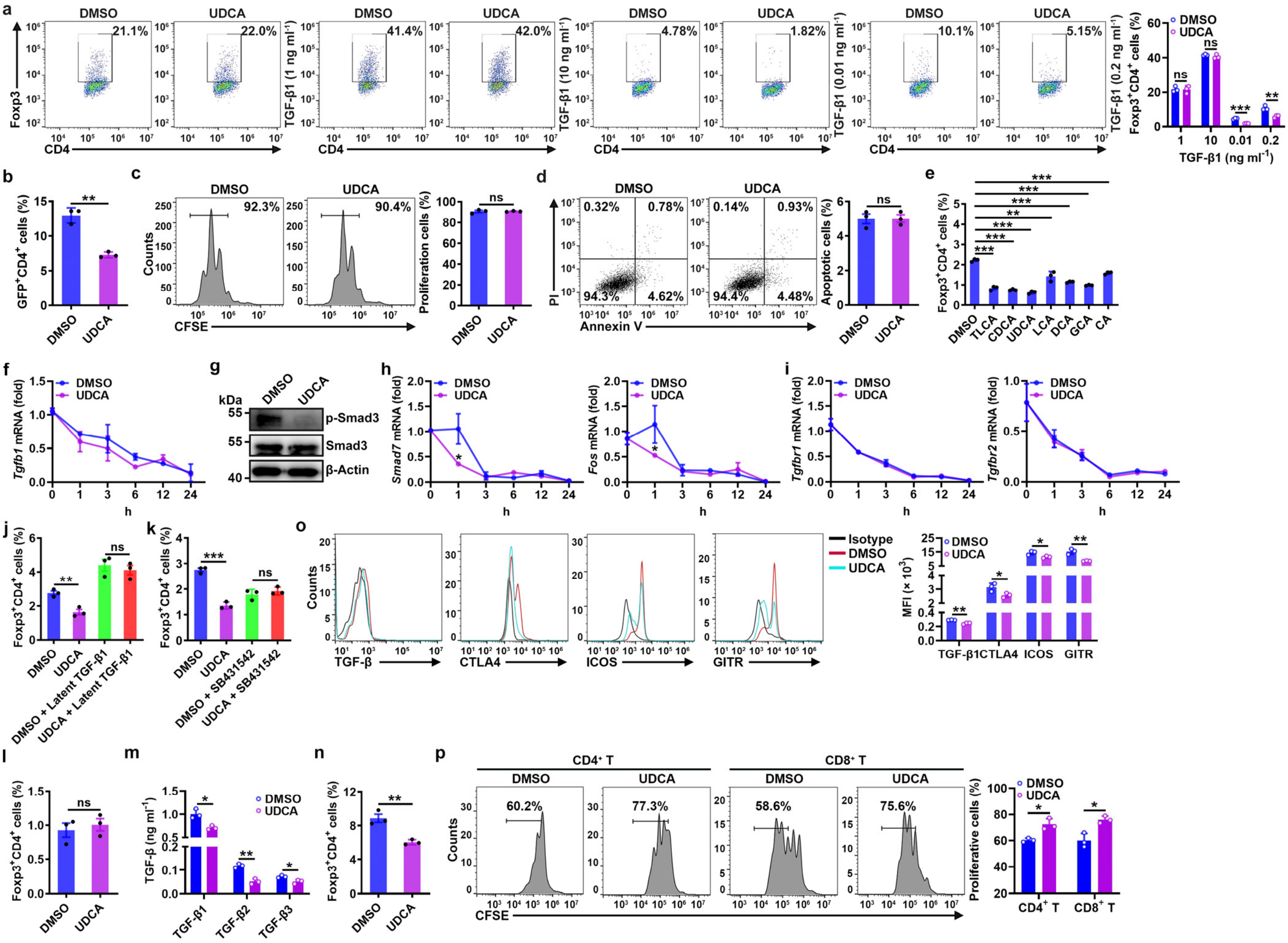

Supplementary Figure 3 Reduced TGF-β contributes to UDCA-mediated inhibition of Treg cell differentiation and function. **a**, **b** FC analysis of Foxp3$^+$CD4$^+$ T (**a**) or GFP$^+$CD4$^+$ T (**b**) cells in naïve CD4$^+$ T cells from wild-type (WT) (**a**) or *Foxp3^{GFP}* (**b**) mice stimulated with anti-CD3, anti-CD28, and 50 μM UDCA, as well as the indicated concentration of TGF-β1 (**a**) or 0.2 ng/ml TGF-β1 in serum-free medium (**b**) for 3 days. **c**, **d** FC analysis of proliferation (**c**) and apoptosis (**d**) of naïve CD4$^+$ T cells stimulated with anti-CD3, anti-CD28, and 50 μM UDCA for 4 days. **e** FC analysis of Foxp3$^+$CD4$^+$ T cells in naïve CD4$^+$ T cells stimulated with anti-CD3 and anti-CD28 in the presence of 50 μM taurolithocholic acid (TLCA), chenodeoxycholic acid (CDCA), UDCA, lithocholic acid (LCA), deoxycholic acid (DCA), glycocholic acid (GCA) or cholic acid (CA) for 4 days. **f** Real-time PCR analysis of the *Tgfb1* mRNA in naïve CD4$^+$ T cells stimulated with anti-CD3, anti-CD28, and 50 μM UDCA for the indicated times. **g-i** IB analysis of

the indicated proteins (**g**) or real-time PCR analysis of the indicated mRNAs (**h**, **i**) in naïve $CD4^+$ T cells stimulated with anti-CD3 and anti-CD28 in the presence of 50 μM UDCA for 24 h (**g**) or for the indicated time (**h**, **i**). **j**, **k** FC analysis of $Foxp3^+CD4^+$ T cells in naïve $CD4^+$ T cells stimulated with anti-CD3, anti-CD28 and 50 μM UDCA, as well as 2 ng latent TGF-β1 (**j**) or 0.5 μM ALK5 inhibitor (SB431542) (**k**) for 4 days. **l** FC analysis of $Foxp3^+CD4^+$ T cells in naïve $CD4^+$ T cells from *Tgfbr2*$^{f/f}$*Er-cre* mice stimulated with anti-CD3, anti-CD28, and 50 μM UDCA for 4 days. **m**, ELISA analysis of TGF-β1, TGF-β2 and TGF-β3 proteins in LLC-OVA cells treated with 50 μM UDCA for 24 h. **n** FC analysis of $Foxp3^+CD4^+$ T cells in naïve $CD4^+$ T cells from OT-II mice cocultured with LLC-OVA cells with 50 μM UDCA in serum-free medium for 4 days. **o** FC analysis of TGF-β, CTLA-4, ICOS, and GITR on Treg cells induced by supernatant from UDCA-treated LLC-OVA. **p** FC analysis of the proliferation of effector $CD4^+$ T and $CD8^+$ T cells cocultured with GFP^+ Treg cells induced by anti-CD3, anti-CD28, and supernatants from LLC-OVA treated with or without 50 μM UDCA at a ratio of 4:1 for 3 days. Representative results from two (**c**, **d**, **f**, **h**, **i**) or three (**a**, **b**, **e**, **g**, **j-p**) independent experiments are shown ($n = 3$ in all statistical groups). *$P < 0.05$; **$P < 0.01$; ***$P < 0.001$ and ns, not significant (unpaired two-tailed Student's *t*-test; mean and s.d.). See Source Data file for the exact *P*-values.

Supplementary Figure 4

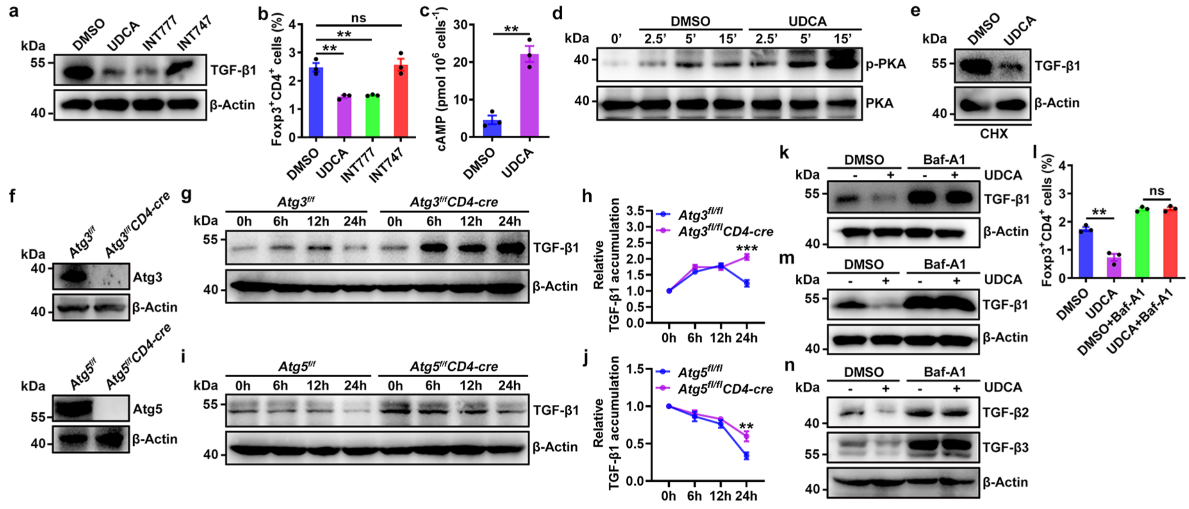

Supplementary Figure 4 UDCA induces autophagy-dependent degradation of TGF-β via the TGR5-cAMP-PKA axis. **a**, **b** IB analysis of TGF-β1 (**a**) and FC analysis of Foxp3$^+$CD4$^+$ T cells (**b**) in 50 μM UDCA-, TGR5 agonist INT777- or FXR agonist INT747-treated naïve CD4$^+$ T cells stimulated with anti-CD3, anti-CD28 for 24 h (**a**) or 4 days (**b**). **c**, **d** cAMP levels (**c**) and IB analysis of p-PKA (**d**) in naïve CD4$^+$ T cells stimulated with anti-CD3, anti-CD28 and 50 μM UDCA for 24 h (**c**) or the indicated time (**d**). **e** IB analysis of TGF-β1 in naïve CD4$^+$ T cells stimulated with anti-CD3, anti-CD28 and 50 μM UDCA along with 5 μM CHX for 8 h. **f-j** IB analysis of Atg3, Atg5 (**f**) or TGF-β1 (**g**, **i**) and statistical analysis of TGF-β1 stability (**h**, **j**) in Atg3- (**f**, **g**, **h**)- or Atg5 (**f**, **i**, **j**)-deficient naïve CD4$^+$ T cells without (**f**) or with (**g-j**) anti-CD3 and anti-CD28 stimulation for the indicated times. **k**, **l** IB analysis of TGF-β1 (**k**) and FC analysis of Foxp3$^+$CD4$^+$ T cells (**l**) in naïve CD4$^+$ T cells stimulated with anti-CD3, anti-CD28 and 50 μM UDCA with or without 1 nM Baf-A1 for 24 h (**k**) or for 4 days (**l**). **m** IB analysis of TGF-β1 in HEK293T cells (overexpressing exogenous TGF-β1) treated with 50 μM UDCA with or without 10 nM Baf-A1 for 24 h. **n** IB analysis of TGF-β2 and TGF-β3 in naïve CD4$^+$ T cells stimulated with anti-CD3, anti-CD28 and 50 μM UDCA with or without 1 nM Baf-A1 for 24 h. All representative results from two (**f**) or three (**a-e**, **g-n**) independent experiments are shown (n = 3 in all statistical groups). **$P < 0.01$; ***P

< 0.001 and ns, not significant (unpaired two-tailed Student's *t*-test; mean and s.d.). See Source Data file for the exact *P*-values.

Supplementary Figure 5

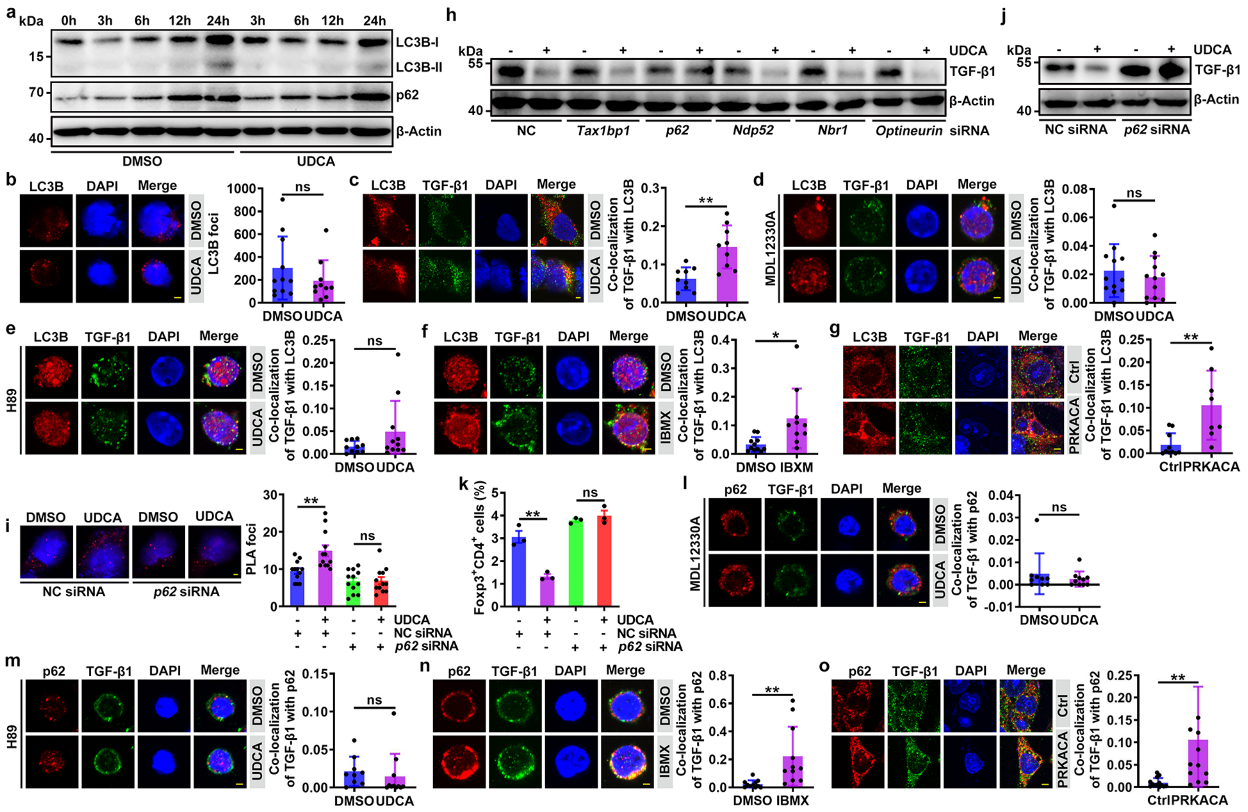

Supplementary Figure 5 UDCA induces p62-dependent sorting of TGF-β into autophagosomes. **a**, **b** IB analysis of LC3B and p62 (**a**) or immunofluorescence analysis of LC3B (**b**) in naïve $CD4^+$ T cells stimulated with anti-CD3, anti-CD28, and 50 μM UDCA for the indicated time (**a**) or for 24 h (**b**). **c-g** Immunofluorescence analysis of LC3B and TGF-β1 HEK293T cells stimulated with 50 μM UDCA (**c**) or in naïve $CD4^+$ T cells stimulated with anti-CD3, anti-CD28 and 50 μM UDCA, as well as 5 μM MDL12330A (an inhibitor of adenylate cyclase) (**d**) or 2 μM H89 (an inhibitor of PKA) (**e**), or in naïve $CD4^+$ T cells stimulated with anti-CD3, anti-CD28 and 100 μM IBMX treatment (**f**) and in NIH-3T3 cells with PRKACA overexpression (**g**) for 24 h. **h** IB analysis of TGF-β1 in UDCA-treated NIH-3T3 cells silenced with the indicated mRNA. **i** Confocal microscopy analysis of PLA^+ spots showing the interaction between TGF-β1 and LC3B in HEK293 cells transfected with NC or *p62* siRNA followed by DMSO or 50 μM UDCA treatment for 24 h. **j**, **k** IB analysis of TGF-β1 (**j**) and FC analysis of $Foxp3^+CD4^+$ cells (**k**)

in p62-knockdown naïve $CD4^+$ T cells stimulated with anti-CD3, anti-CD28 and 50 μM UDCA for 24 h (**j**) and 4 days (**k**). **l-o** Immunofluorescence analysis of p62 and TGF-β1 in naïve $CD4^+$ T cells stimulated with anti-CD3, anti-CD28 and 50 μM UDCA, as well as 5 μM MDL12330A (**l**) or 2 μM H89 (**m**), in naïve $CD4^+$ T cells stimulated with anti-CD3, anti-CD28 and 100 μM IBMX treatment (**n**) and in NIH-3T3 cells with PRKACA overexpression (**o**) for 24 h. NC, negative control. Scale bar, 2 μm. All representative results from three independent experiments are shown (n = 11 (DMSO) or 10 (UDCA) in **b**; n = 9 in **c**; n = 12 in **d**; n = 10 (DMSO) or 11 (UDCA) in **e**; n = 11 (DMSO) or 10 (IBMX) in **f**; n = 10 (Ctrl) or 8 (PRKACA) in **g**; n = 12 in **i**; n = 3 in **k**; n = 9 (DMSO) or 10 (UDCA) in **l**; n = 9 (DMSO) or 11 (UDCA) in **m**; n = 11 in **n**; n = 10 (Ctrl) or 13 (PRKACA) in **o**). $*P < 0.05$; $**P < 0.01$ and ns, not significant (unpaired two-tailed Student's t-test; mean and s.d.). See Source Data file for the exact P-values.

Supplementary Figure 6

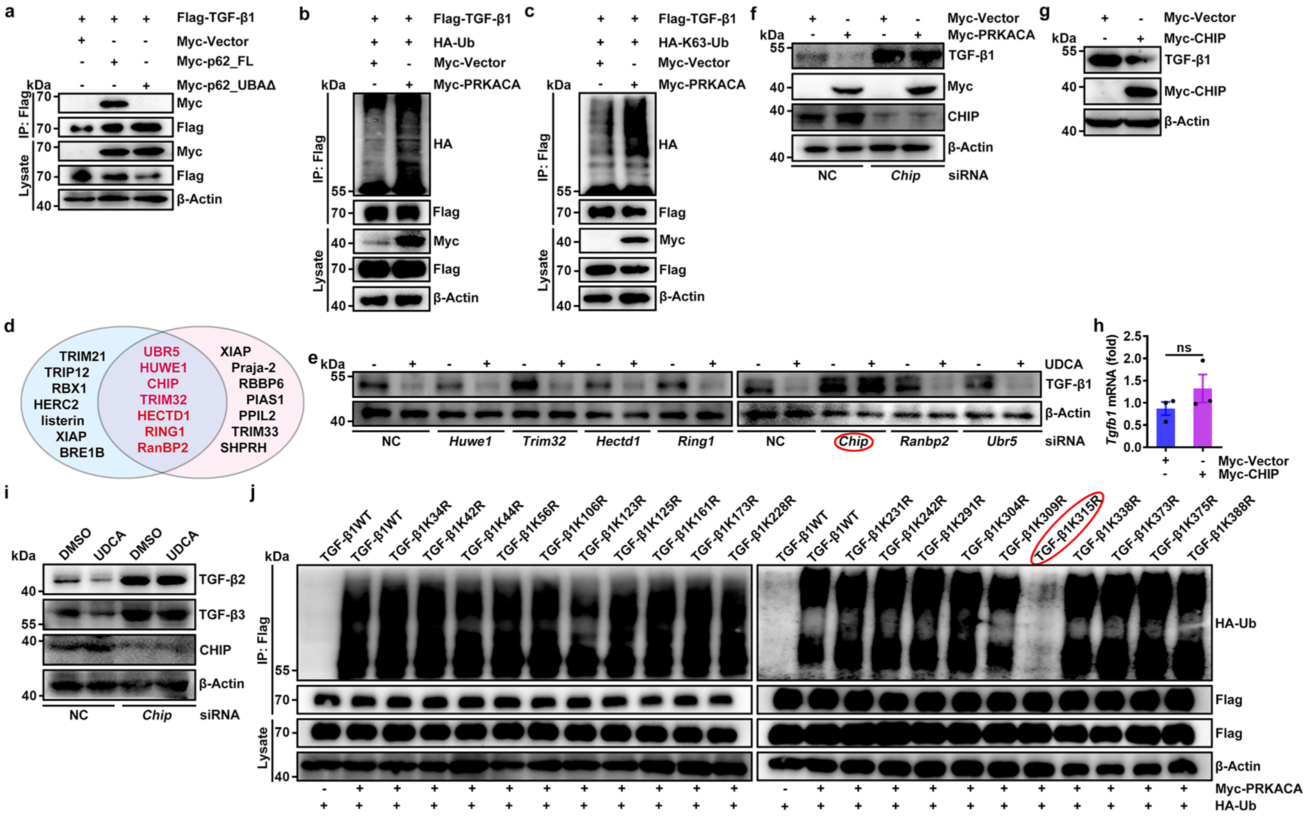

Supplementary Figure 6 CHIP mediates K63-linked ubiquitination of TGF-β1 at K315. **a** IB analysis of p62 and TGF-β1 in lysates of HEK293T cells transfected with vectors expressing Flag-TGF-β1 and Myc-p62_FL or Flag-TGF-β1 and Myc-p62_UBAΔ for 24 h assessed after IP with anti-Flag. **b**, **c** IB analysis of total (**b**) or K63-linked (**c**) ubiquitination of TGF-β1 in lysates of HEK293T cells transfected with vectors expressing Flag-TGF-β1 and HA-Ub, with or without vectors expressing Myc-PRKACA for 24 h assessed after IP with anti-Flag. **d** E3 ligases interacting with TGF-β1 (blue) or PKA (pink) and TGF-β1 and PKA (purple). **e** IB analysis of TGF-β1 in NIH-3T3 cells transfected with the indicated siRNA for 24 h and then treated with 50 μM UDCA for another 24 h. **f** IB analysis of TGF-β1 in CHIP-knockdown NIH-3T3 cells transfected with or without vectors expressing Myc-PRKACA for 24 h. **g, h** IB (**g**) or real-time PCR (**h**) analysis of TGF-β1 in HEK293T cells transfected with or without vectors expressing Myc-CHIP for 24 h. **i** IB analysis of TGF-β2 and TGF-β3 in CHIP-knockdown naïve CD4^{+} T cells stimulated with anti-CD3, anti-CD28 and 50 μM UDCA for 24 h. **j** IB analysis of TGF-β1 ubiquitination in HEK293T

cells transfected with vectors expressing the indicated TGF-β1 mutants with Flag tag and vectors expressing Myc-PRKACA and HA-Ub for 24 h assessed after IP with anti-Flag. All representative results from three **(a-c**, **e-j)** independent experiments are shown (*n* = 3 in **h**). NC, negative control. ns, not significant (unpaired two-tailed Student's *t*-test; mean and s.d.).

Supplementary Figure 7

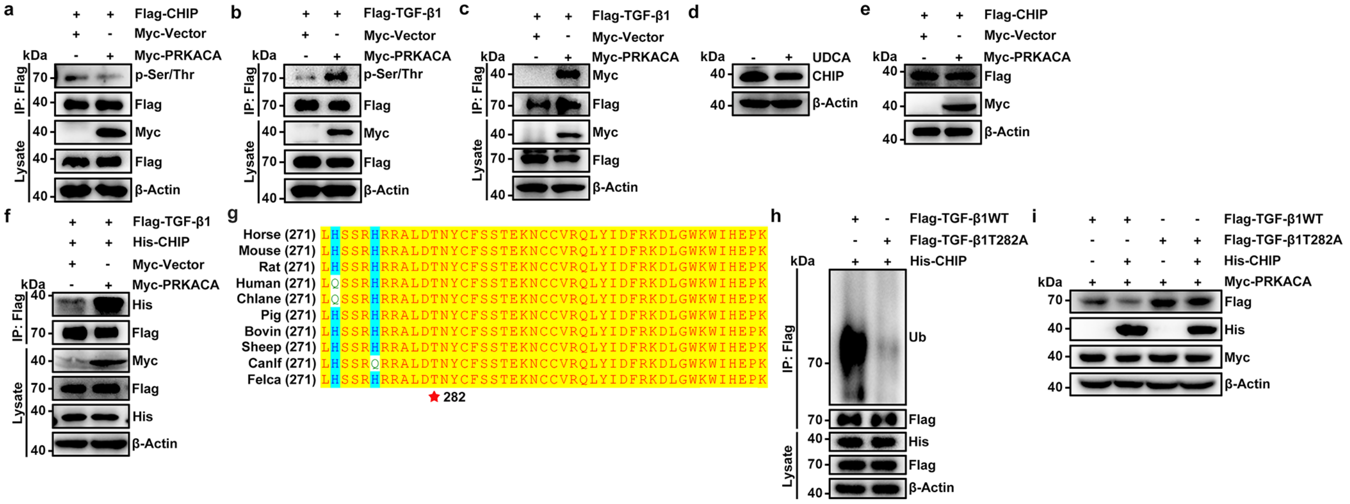

Supplementary Figure 7 TGF-β1 phosphorylated by PKA has an enhanced ability to bind CHIP. **a**, **b** IB analysis of p-Ser/Thr of CHIP (**a**) or TGF-β1 (**b**) in lysates of HEK293T cells transfected with vectors expressing Flag-CHIP (**a**) or Flag-TGF-β1 (**b**) as well as vectors expressing Myc-PRKACA for 24 h assessed after IP with anti-Flag. **c** IB analysis of PRKACA in lysates of HEK293T cells transfected with vectors expressing Flag-TGF-β1 and Myc-PRKACA for 24 h assessed after IP with anti-Flag. **d**, **e** IB analysis of CHIP in HEK293T cells treated with 50 μM UDCA (**d**) or transfected with vectors expressing Flag-CHIP and Myc-PRKCA (**e**) for 24 h. **f** IB analysis of CHIP in lysates of HEK293T cells transfected with vectors expressing Flag-TGF-β1 and His-CHIP, as well as vectors expressing Myc-PRKACA for 24 h assessed after IP with anti-Flag. **g** Alignment of TGF-β1 orthologs. Asterisk, Thr 282. **h** IB analysis of ubiquitinated TGF-β1 in HEK293T cells transfected with vectors expressing His-CHIP and Flag-TGF-β1WT or Flag-TGF-β1T282A assessed after IP with anti-Flag. **i** IB analysis of TGF-β1 in HEK293T cells transfected with vectors expressing His-CHIP, Myc-PRKACA and Flag-TGF-β1WT or Flag-TGF-β1T282A. All representative results from three **(a-f**, **h**, **i)** independent experiments are shown.

Supplementary Figure 8

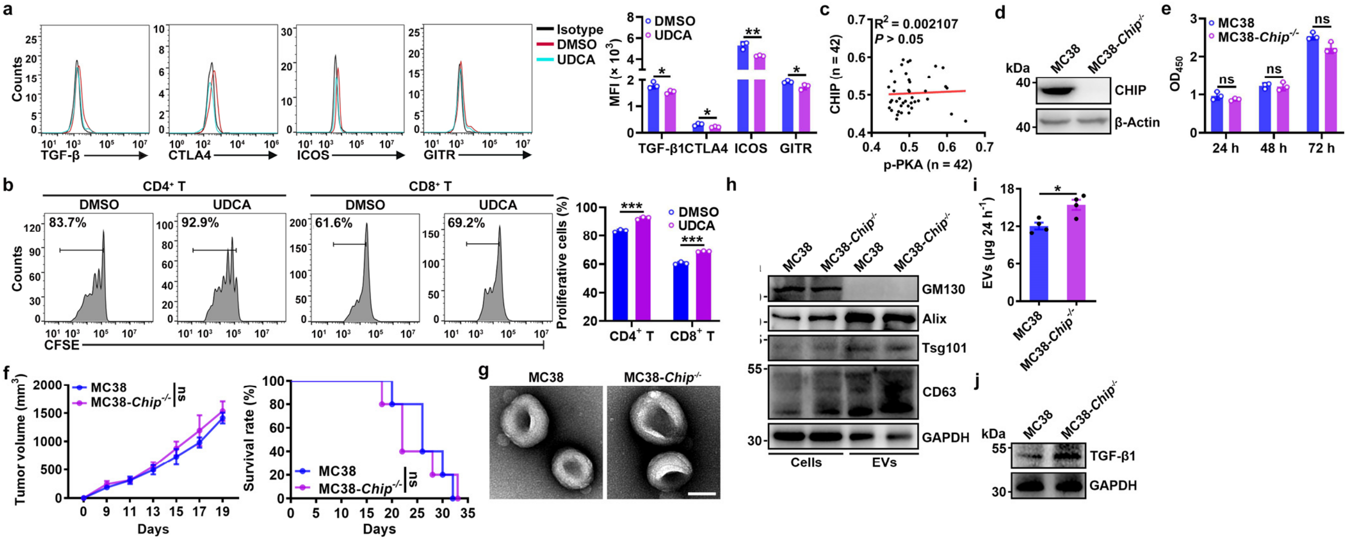

Supplementary Figure 8 CHIP is negatively associated with tumor progression by reducing TGF-β. **a** FC analysis of TGF-β1, CTLA4, ICOS, and GITR of human Treg cells induced by anti-CD3 and anti-CD28 stimulation for 4 days cultured in supernatants of A549 cells prestimulated with 50 μM UDCA for 24 h. **b** FC analysis of proliferation of CFSE-labeled $CD4^+$ or $CD8^+$ T cells cocultured with $CD4^+CD25^+$ Treg cells obtained in **a** at 4:1 ratio in anti-CD3 and anti-CD28-coated plates for 5 days. **c** Correlation between p-PKA and CHIP protein levels. **d** IB detection of CHIP in MC38 and MC38-*Chip*$^{-/-}$ cells. **e** CCK-8 analysis of the viability of MC38 and MC38-*Chip*$^{-/-}$ cells. **f** Tumor sizes and mouse survival of MC38 and MC38-*Chip*$^{-/-}$ tumor-bearing nude mice. **g** Electron microscope detection of morphology of EVs from MC38 and MC38-*Chip*$^{-/-}$ cells. Scale bar, 100 nm. **h** IB detection of the indicated proteins in MC38 and MC38-*Chip*$^{-/-}$ cells, and in EVs from these cells. **i** BCA measurement of protein amount of EVs from 1×10^7 MC38 and MC38-*Chip*$^{-/-}$ cells. **j** IB detection of TGF-β1 in EVs from MC38 and MC38-*Chip*$^{-/-}$ cells. Representative results from two (**e**, **g**, **h**) or three (**a**, **b**, **d**, **f**, **i**, **j**) independent experiments are shown (n = 3 in **a**, **b**, **e**, **i**; n = 5 in **f**). *P < 0.05; **P < 0.01; ***P < 0.001; ns, not significant (unpaired two-tailed Student's t-test except log-rank test was used for survival rate analysis; mean and s.d.). See Source Data file for the exact P-values.

Shen et al.

Supplementary Figure 9

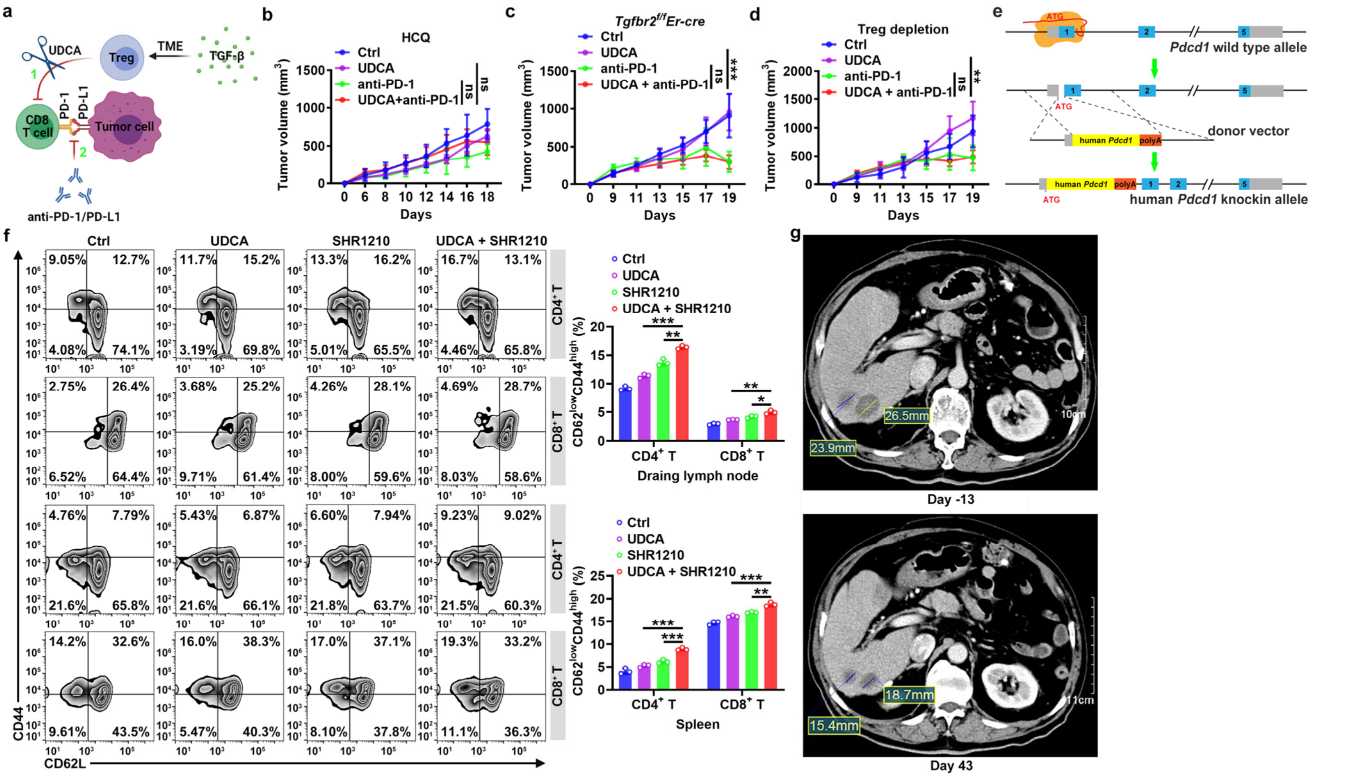

Supplementary Figure 9 UDCA enhances the antitumor effects of anti-PD-1. **a** Putative benefits in release of tumor immunosuppression by UDCA and anti-PD-1 or anti-PD-L1 combinatorial therapy. TME, tumor microenvironment. **b** Tumor sizes of LLC tumor-bearing mice received oral administration of 30 mg kg^{-1} UDCA every other day along with intravenous injection with 50 μg anti-PD-1 every 4 days and i.p. injection with 60 mg kg^{-1} HCQ every other day. **c** Tumor sizes of LLC tumor-bearing *Tgfbr2^f/f^Er-cre* mice that received oral administration of 30 mg kg^{-1} UDCA every other day along with intravenous injection with 50 μg anti-PD-1 every 4 days. **d** Tumor sizes of LLC tumor-bearing mice received oral administration of 30 mg kg^{-1} UDCA every other day along with intravenous injection with 50 μg anti-PD-1 every 4 days and i.p. injection with 100 μg anti-CD25 every 3 days. **e** Construction strategy of humanized PD-1 mice. **f** FC analysis on day 33 of memory T cells in draining lymph nodes and spleen of MC38-huPD-L1 tumor-bearing mice received oral administration of 30 mg kg^{-1} UDCA every other day along with intravenous injection with 50 μg SHR1210 every 4 days. **g** Computed tomography examination on

days -13 and 43 of a pancreatic cancer patient who received anti-PD-1 and UDCA treatment. The day on which the patient received anti-PD-1 treatment was defined as day 0. Representative results from two independent experiments are shown ($n = 5$ in **b-d**; $n = 3$ in **f**). *$P < 0.05$; **$P < 0.01$; ***$P < 0.001$ and ns, not significant (unpaired two-tailed Student's *t*-test; mean and s.d.). See Source Data file for the exact *P*-values.

Supplementary Figure 10

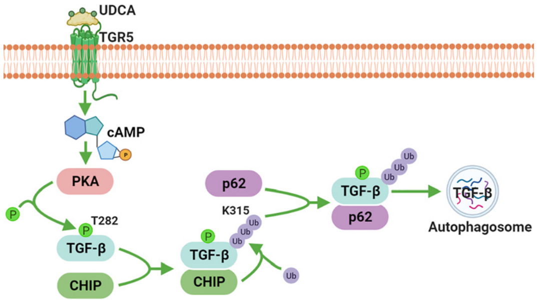

Supplementary Figure 10 The working model of UDCA-mediated TGF-β degradation. UDCA-mediated ligation of TGR5 enhanced intracellular cAMP levels, resulting in activation of PKA. Subsequently, PKA phosphorylated TGF-β at the T282 site, leading to the recruitment of CHIP. Then, CHIP ubiquitinated TGF-β, initiating autophagy sorting and subsequent degradation of TGF-β.

Supplementary Figure 11

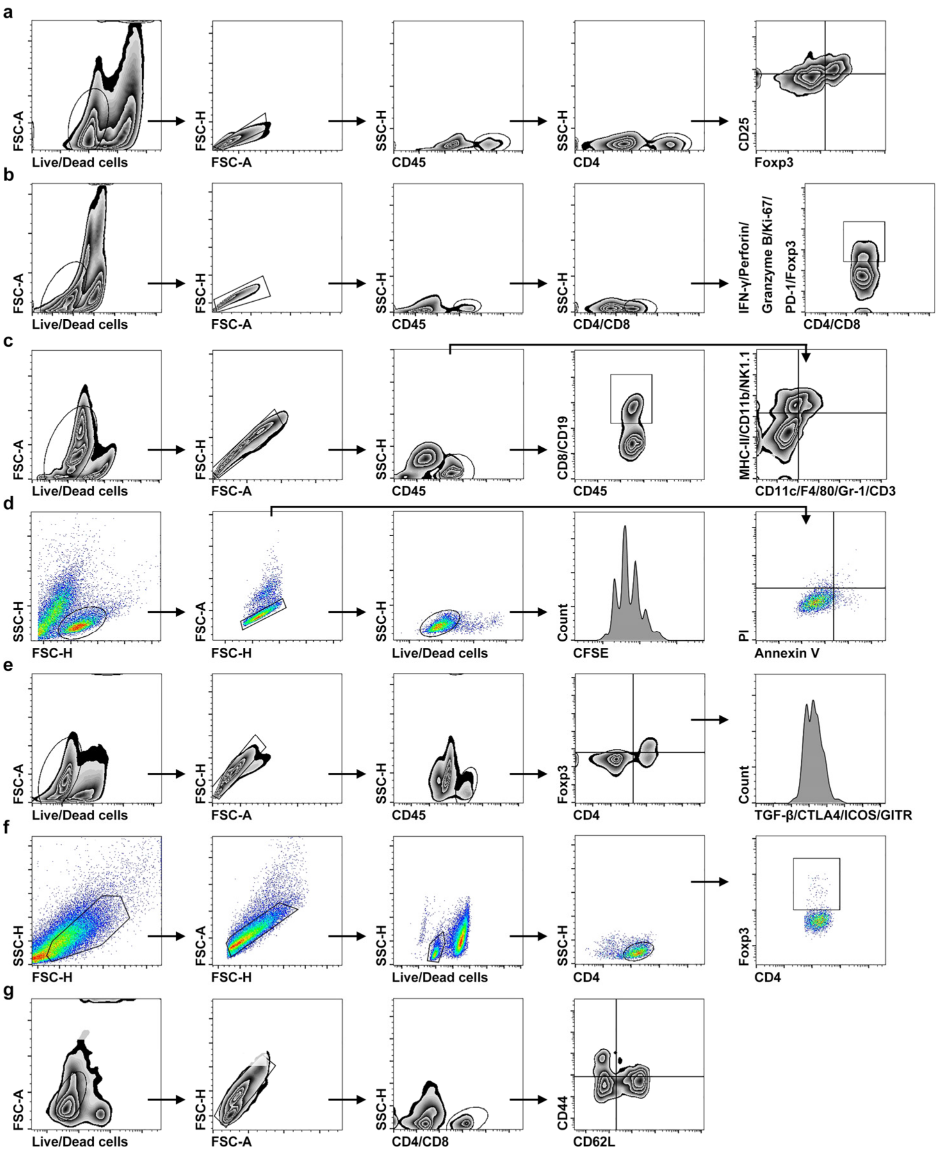

Supplementary Figure 11 Gating strategies of FC analyses. (**a-f**) Gating strategies of Fig. 1b, 1h and Supplementary Fig. 1g (**a**), Fig. 1b, 1e, 6p, 7c and Supplementary Fig. 2c (**b**), Fig. 1b, 1d, 1h, 7c and Supplementary Fig. 1b, 1g (**c**), Fig. 1i and Supplementary Fig. 2f, 3c, 3d, 3p, 8b (**d**), Supplementary Fig. 2a, 2b, 2e, 3o, 8a (**e**), Fig. 2a, 2c, 2e, 2i, 3b, 3e, 3f, 3i, 3j, 3r, 3s, 4d, 5i, 6a, 6c, and Supplementary Fig. 3a, 3b, 3e, 3j, 3k, 3l, 3n, 4b, 4l, 5k (**f**), Fig. 7d, and Supplementary Fig. 9f (**g**).

Supplementary Table 1: Basic information of NSCLC patients.

Characteristics	NSCLC patients
Number	48
Age (years)	
≤ 30	0
30-50	7
≥ 50	41
Gender	
Male	29
Female	19
Pathological types	
Adenocarcinoma	27
Squamous cell carcinoma	21
clinical Stage	
I	10
II	13
III	22
IV	3

Supplementary Table 2: Information of patients with ICI treatment.

No.	A	G	PT	S	D-ICI	N-ICI	T-ICI	D-US	D-UE
**Lung cancer**									
1	52	F	AC	IV	2016/1	Nivolumab	PD-1	2016/3	2016/5
2	54	F	SCC	IV	2018/6	Nivolumab	PD-1	2018/10	2018/11
3	54	M	SCC	IV	2019/11	Sintilimab	PD-1	2019/10	2019/10
4	60	F	AC	IV	2019/10	Atezolizumab	PD-L1	2019/10	2019/10
5	63	M	AC	IV	2019/6	Tislelizumab	PD-1	2019/10	2019/10
6	56	M	SCC	IV	2019/8	Camrelizumab	PD-1	2019/7	2019/8
7	69	M	SCC	IV	2017/10	Nivolumab	PD-1	2017/10	2018/2
8	66	F	NSCLC	IV	2019/4	Camrelizumab	PD-1		
9	72	M	AC	IV	2019/5	Tislelizumab	PD-1		
10	60	F	SCC	IV	2019/5	Nivolumab	PD-1		
11	63	F	AC	IIIB	2019/7	Atezolizumab	PD-L1		
12	58	M	AC	IV	2017/12	Durvalumab	PD-L1		
13	69	M	NSCLC	IV	2018/11	Camrelizumab	PD-1		
14	63	M	AC	IV	2018/11	Camrelizumab	PD-1		
15	66	M	AC	IV	2019/4	Sintilimab	PD-1		
16	62	M	SCC	IV	2019/4	Toripalimab	PD-1		
17	55	F	NSCLC	IV	2019/2	Nivolumab	PD-1		
18	61	M	SCC	IV	2018/10	Nivolumab	PD-1		
19	67	M	SCC	IV	2019/5	Sintilimab	PD-1		
20	56	F	NSCLC	IV	2019/1	Nivolumab	PD-1		
21	62	M	AC	IV	2018/12	Nivolumab	PD-1		
22	53	M	AC	IV	2018/4	Permbrolizumab	PD-1		
23	33	M	AC	IV	2019/3	Permbrolizumab	PD-1		
24	72	M	AC	IV	2018/11	Nivolumab	PD-1		
25	70	F	AC	IV	2018/8	Permbrolizumab	PD-1		
26	56	F	AC	IV	2019/4	Tislelizumab	PD-1		
27	66	M	AC	IV	2019/3	Tislelizumab	PD-1		
28	74	F	AC	IV	2019/3	Sintilimab	PD-1		
29	58	M	SCC	IV	2019/3	Tislelizumab	PD-1		
30	61	M	SCC	IV	2019/2	Toripalimab	PD-1		
31	72	F	AC	IV	2019/4	Permbrolizumab	PD-1		
32	53	M	AC	IV	2018/11	Nivolumab	PD-1		
33	63	M	SCC	IV	2018/2	Permbrolizumab	PD-1		
34	54	M	AC	IV	2018/9	Nivolumab	PD-1		
35	75	M	SCC	IV	2018/11	Nivolumab	PD-1		
36	61	M	SCC	IV	2019/3	Tislelizumab	PD-1		
37	62	M	AC	IV	2019/3	Durvalumab	PD-L1		

38	68	M	SCC	IV	2018/4	Durvalumab	PD-L1		
39	60	M	AC	IV	2019/3	Tislelizumab	PD-1		
40	51	M	AC	IV	2019/3	Permbrolizumab	PD-1		
41	58	M	AC	IV	2018/10	Tislelizumab	PD-1		
42	65	M	SCC	IV	2019/2	Sintilimab	PD-1		
43	64	F	AC	IV	2019/1	Permbrolizumab	PD-1		
44	72	M	SCC	IV	2019/1	Sintilimab	PD-1		
45	59	M	SCC	IIIB	2019/3	Toripalimab	PD-1		
46	64	F	AC	IV	2018/11	Durvalumab	PD-1		
47	55	F	AC	IV	2018/12	Tislelizumab	PD-1		
48	72	M	SCC	IV	2018/11	Nivolumab	PD-1		
49	54	M	SCC	IIIB	2018/11	Tislelizumab	PD-1		
50	55	F	AC	IV	2018/11	Tislelizumab	PD-1		
51	56	M	NSCLC	IV	2017/11	Permbrolizumab	PD-1		
52	79	M	SCC	IV	2017/5	Permbrolizumab	PD-1		
53	55	M	NSCLC	IV	2017/1	Permbrolizumab	PD-1		
54	62	M	AC	IV	2018/12	Tislelizumab	PD-1		
55	60	M	SCC	IV	2019/1	Sintilimab	PD-1		
56	47	M	AC	IV	2018/10	Durvalumab	PD-1		
57	65	M	NSCLC	IV	2018/10	Nivolumab	PD-1		
58	69	M	AC	IV	2018/11	Permbrolizumab	PD-1		
59	51	M	AC	IV	2018/11	Tislelizumab	PD-1		
60	69	M	NSCLC	IV	2018/11	Tislelizumab	PD-1		
61	69	M	SCC	IIIB	2019/1	Tislelizumab	PD-1		
62	56	F	AC	IV	2019/2	Nivolumab	PD-1		
63	69	M	SCC	IV	2019/6	Sintilimab	PD-1		
64	67	M	SCC	IV	2019/5	Permbrolizumab	PD-1		
65	50	F	AC	IIIB	2019/6	Toripalimab	PD-1		
66	60	M	AC	IV	2019/3	Toripalimab	PD-1		
67	51	F	AC	IV	2019/4	Toripalimab	PD-1		
68	73	M	AC	IV	2019/5	Sintilimab	PD-1		
69	69	M	SCC	IIIB	2019/3	Toripalimab	PD-1		
70	65	F	AC	IV	2019/5	Tislelizumab	PD-1		
71	68	M	SCC	IV	2019/5	Nivolumab	PD-1		
72	64	M	NSCLC	IV	2019/2	Atezolizumab	PD-L1		
73	44	F	AC	IV	2018/7	Permbrolizumab	PD-1		
74	74	M	AC	IV	2018/12	Camrelizumab	PD-1		
75	71	F	AC	IV	2019/4	Sintilimab	PD-1		
76	64	M	SCC	IIIB	2019/5	Permbrolizumab	PD-1		
77	61	M	SCC	IV	2017/11	Permbrolizumab	PD-1		
78	37	F	AC	IIIB	2018/12	Atezolizumab	PD-L1		

79	56	M	SCC	IV	2019/5	Nivolumab	PD-1		
80	66	M	SCC	IV	2019/7	Atezolizumab	PD-L1		
81	70	M	SCC	IV	2019/7	Nivolumab	PD-1		
82	62	M	AC	IV	2018/12	Tislelizumab	PD-1		
83	64	M	NSCLC	IV	2019/4	Tislelizumab	PD-1		
84	57	M	SCC	IV	2019/3	Sintilimab	PD-1		
85	55	M	SCC	IV	2019/4	Nivolumab	PD-1		
86	66	M	SCC	IV	2019/6	Toripalimab	PD-1		
87	82	M	AC	IV	2019/5	Sintilimab	PD-1		
88	53	M	AC	IV	2019/4	Tislelizumab	PD-1		
89	57	M	AC	IIIB	2019/6	Atezolizumab	PD-L1		
90	55	M	AC	IIIB	2019/6	Permbrolizumab	PD-1		
91	59	M	AC	IV	2019/3	Tislelizumab	PD-1		
92	67	M	SCC	IIIB	2019/4	Tislelizumab	PD-1		
93	68	M	NSCLC	IV	2019/3	Toripalimab	PD-1		
94	56	M	AC	IV	2019/7	Sintilimab	PD-1		
95	49	M	AC	IV	2019/2	Toripalimab	PD-1		
96	64	F	AC	IV	2019/3	Sintilimab	PD-1		
97	67	M	SCC	IV	2019/4	Toripalimab	PD-1		
98	45	M	AC	IV	2019/1	Tislelizumab	PD-1		
99	64	M	SCC	IV	2019/6	Sintilimab	PD-1		
100	71	M	SCC	IV	2019/6	Sintilimab	PD-1		
101	56	M	SCC	IV	2018/4	Permbrolizumab	PD-1		
102	50	M	SCC	IV	2016/4	Nivolumab	PD-1		
103	61	M	SCC	IV	2017/9	Camrelizumab	PD-1		
104	68	F	AC	IV	2017/1	Atezolizumab	PD-L1		
105	59	M	AC	IV	2018/2	Tislelizumab	PD-1		
106	45	F	AC	IV	2016/12	Atezolizumab	PD-L1		
107	61	M	NSCLC	IV	2018/12	Permbrolizumab	PD-1		
108	64	M	NSCLC	IV	2018/11	Permbrolizumab	PD-1		
109	44	M	AC	IV	2016/11	Atezolizumab	PD-L1		
110	67	M	AC	IV	2018/5	Tislelizumab	PD-1		
111	51	F	AC	IV	2018/7	Tislelizumab	PD-1		
112	65	M	SCC	IV	2018/10	Nivolumab	PD-1		
113	55	M	AC	IV	2018/8	Nivolumab	PD-1		
114	56	M	AC	IV	2018/6	Nivolumab	PD-1		
115	60	M	SCC	IV	2018/9	Nivolumab	PD-1		
116	60	M	AC	IV	2018/10	Permbrolizumab	PD-1		
117	64	F	AC	IIIB	2018/10	Tislelizumab	PD-1		
118	64	M	AC	IV	2018/7	Nivolumab	PD-1		
119	60	M	NSCLC	IV	2018/8	Nivolumab	PD-1		

120	74	M	SCC	IV	2016/5	Nivolumab	PD-1		
121	63	F	AC	IV	2016/3	Nivolumab	PD-1		
122	60	M	SCC	IV	2018/11	Permbrolizumab	PD-1		
123	59	M	AC	IV	2017/3	Atezolizumab	PD-L1		
124	70	M	AC	IV	2016/9	Nivolumab	PD-1		
125	64	M	AC	IV	2016/5	Nivolumab	PD-1		
126	67	M	SCC	IV	2018/6	Permbrolizumab	PD-1		
127	63	M	SCC	IV	2018/9	Nivolumab	PD-1		
128	55	M	SCC	IV	2017/1	Atezolizumab	PD-L1		
129	58	M	AC	IV	2016/12	Atezolizumab	PD-L1		
130	47	M	AC	IV	2018/9	Sintilimab	PD-1		
131	59	M	SCC	IIIB	2018/9	Nivolumab	PD-1		
132	60	M	NSCLC	IV	2018/10	Nivolumab	PD-1		
133	50	M	AC	IV	2018/4	Tislelizumab	PD-1		
134	49	F	AC	IV	2018/7	Nivolumab	PD-1		
135	64	F	AC	IV	2018/9	Nivolumab	PD-1		
136	58	M	SCC	IV	2016/4	Nivolumab	PD-1		
137	63	M	SCC	IV	2017/4	Atezolizumab	PD-L1		
138	74	M	SCC	IV	2016/9	Nivolumab	PD-1		
139	73	M	AC	IV	2018/10	Permbrolizumab	PD-1		
140	62	M	SCC	IV	2017/10	Camrelizumab	PD-1		
141	56	M	SCC	IV	2018/3	Nivolumab	PD-1		
142	68	M	AC	IV	2018/9	Nivolumab	PD-1		
143	63	M	SCC	IV	2018/9	Nivolumab	PD-1		
144	58	F	AC	IV	2017/7	Camrelizumab	PD-1		
145	63	M	SCC	IV	2016/6	Nivolumab	PD-1		
146	61	M	SCC	IV	2017/10	Camrelizumab	PD-1		
147	56	M	AC	IIIB	2018/8	Nivolumab	PD-1		
148	51	F	AC	IV	2018/3	Camrelizumab	PD-1		
149	58	M	SCC	IV	2017/11	Camrelizumab	PD-1		
150	60	F	AC	IV	2018/7	Nivolumab	PD-1		
151	66	M	SCC	IV	2018/3	Tislelizumab	PD-1		
152	67	M	AC	IIIB	2018/2	Tislelizumab	PD-1		
153	59	F	AC	IV	2018/5	Tislelizumab	PD-1		
154	56	M	SCC	IV	2018/8	Tislelizumab	PD-1		
155	58	F	AC	IV	2018/6	Tislelizumab	PD-1		
156	56	F	AC	IV	2018/5	Tislelizumab	PD-1		
157	63	M	SCC	IV	2017/1	Atezolizumab	PD-L1		
158	65	F	AC	IV	2018/7	Tislelizumab	PD-1		
159	65	F	AC	IV	2018/7	Tislelizumab	PD-1		
160	68	M	AC	IV	2018/7	Tislelizumab	PD-1		

161	61	M	SCC	IV	2018/11	Permbrolizumab	PD-1		
162	36	F	AC	IV	2017/10	Camrelizumab	PD-1		
163	51	F	AC	IIIB	2018/3	Tislelizumab	PD-1		
164	71	F	SCC	IV	2018/6	Nivolumab	PD-1		
165	43	M	SCC	IIIB	2017/10	Camrelizumab	PD-1		
166	61	F	AC	IV	2018/3	Permbrolizumab	PD-1		
167	58	M	NSCLC	IV	2018/9	Nivolumab	PD-1		
168	55	M	AC	IV	2018/11	Nivolumab	PD-1		
169	59	M	SCC	IV	2018/9	Nivolumab	PD-1		
170	61	M	AC	IV	2017/1	Permbrolizumab	PD-1		
171	51	F	AC	IIIB	2016/5	Nivolumab	PD-1		
172	52	M	AC	IV	2016/11	Atezolizumab	PD-L1		
173	73	M	SCC	IV	2018/5	Tislelizumab	PD-1		
174	50	M	NSCLC	IV	2018/8	Nivolumab	PD-1		
175	63	M	AC	IV	2018/7	Camrelizumab	PD-1		
176	49	M	AC	IV	2018/6	Tislelizumab	PD-1		
177	70	F	AC	IV	2016/11	Atezolizumab	PD-L1		
178	47	M	SCC	IV	2017/11	Sintilimab	PD-1		
179	63	M	AC	IV	2016/8	Nivolumab	PD-1		
180	62	M	AC	IV	2016/11	Atezolizumab	PD-L1		
181	65	M	AC	IV	2016/11	Atezolizumab	PD-L1		
182	66	M	SCC	IIIB	2018/6	Camrelizumab	PD-1		
183	81	M	NSCLC	IV	2018/10	pembrolizumab	PD-1		
184	57	M	SCC	IIIB	2018/5	Tislelizumab	PD-1		
185	61	M	AC	IV	2018/3	Tislelizumab	PD-1		
186	64	M	AC	IV	2018/1	Camrelizumab	PD-1		
187	69	M	SCC	IIIB	2016/12	Atezolizumab	PD-L1		
188	63	M	AC	IV	2018/7	Nivolumab	PD-1		
189	68	M	SCC	IV	2018/8	Nivolumab	PD-1		
190	47	M	AC	IV	2018/10	Permbrolizumab	PD-1		
191	51	M	SCC	IV	2018/10	Tislelizumab	PD-1		
192	63	M	SCC	IIIB	2018/8	Nivolumab	PD-1		
193	58	M	SCC	IV	2018/7	Tislelizumab	PD-1		
194	44	M	AC	IV	2018/5	Tislelizumab	PD-1		
195	63	M	AC	IV	2018/1	Camrelizumab	PD-1		
196	64	F	AC	IV	2017/12	Camrelizumab	PD-1		
197	63	M	SCC	IV	2018/2	Camrelizumab	PD-1		
198	70	M	SCC	IV	2018/7	Nivolumab	PD-1		
199	53	M	SCC	IV	2018/2	Camrelizumab	PD-1		
200	59	M	SCC	IV	2018/6	Tislelizumab	PD-1		
201	50	M	SCC	IV	2016/11	Atezolizumab	PD-L1		

202	61	M	SCC	IV	2016/12	Atezolizumab	PD-L1		
203	63	M	SCC	IV	2018/3	Tislelizumab	PD-1		
204	67	M	SCC	IV	2017/1	Atezolizumab	PD-L1		
205	65	M	AC	IV	2018/2	Permbrolizumab	PD-1		
206	54	M	AC	IV	2018/1	Tislelizumab	PD-1		
207	54	F	AC	IV	2017/1	Atezolizumab	PD-L1		
208	53	M	SCC	IV	2016/9	Nivolumab	PD-1		
209	64	F	AC	IV	2016/10	Atezolizumab	PD-L1		
210	53	M	AC	IV	2017/8	Camrelizumab	PD-1		
211	63	M	SCC	IIIB	2018/3	Tislelizumab	PD-1		
**Pancreatic cancer**									
1	65	M	SCC	IV	2021/1	MV11 (Maiwei, Shanghai, China)	PD-1	2019/7	continued

Abbreviations: A, Age; G, Gender; PT, Pathological types; S, Stage; D-ICI, Date of ICI treatment start; N-ICI, Name of ICI; T-ICI, Targe of ICI, D-US, Date of Ursofalk treatment start; D-UE, Date of Ursofalk treatment end; AC, Adenosquamous carcinoma; SCC, Squamous cell carcinoma; AC, Adenocarcinoma

Supplementary Table 3: Information of antibodies used in this study.

Supplementary Table 3: The antibodies for FC and IB.			
**Antibodies**	**Source**	**Identifier**	**Dilution ratio**
**fixable viability dye eFluor™ 450**	ThermoFisher	65-0863-14	1:500
**fixable viability dye eFluor™ 520**	ThermoFisher	65-0867-14	1:500
**PB anti-CD45**	BioLegend	103125	1:500
**PE-cy5 anti-CD19**	BioLegend	115510	1:500
**PE anti-CD4**	ThermoFisher	12-0041-82	1:500
**APC anti-CD8**	BioLegend	100712	1:500
**PE anti-Granzyme B**	ThermoFisher	12-8898-82	1:500
**PE anti-IFN-γ**	ThermoFisher	12-7311-82	1:500
**PE anti-ST2**	BioLegend	146607	1:500
**PE anti-Perforin**	BioLegend	154405	1:500
**APC anti-CD11c**	BioLegend	117310	1:500
**PE anti-MHC II**	ThermoFisher	12-5321-82	1:500
**PE anti-CD11b**	ThermoFisher	12-0112-83	1:500
**APC anti-F4/80**	BioLegend	123115	1:500
**APC anti-CD3**	BioLegend	100235	1:500
**PE anti-NK1.1**	BioLegend	108707	1:500
**APC anti-Foxp3**	ThermoFisher	17-5773-82	1:500
**APC anti-Gr1**	ThermoFisher	17-5931-82	1:500
**PE anti-human/mouse Ki-67**	BioLegend	151209	1:500
**PE anti-mouse CD152 (CTLA4)**	BioLegend	106305	1:500
**PE anti-mouse CD278 (ICOS)**	ThermoFisher	12-9942-81	1:500
**PE anti-human CD152 (CTLA4)**	BioLegend	369603	1:500
**PE anti-human CD357 (GITR)**	ThermoFisher	12-5875-41	1:500
**PE anti-TGF-1/-2/-3**	R&D	IC1835P	1:500
**PE anti-CD25**	ThermoFisher	12-0251-83	1:500
**APC anti-CD44**	ThermoFisher	17-0441-83	1:500
**PE anti-CD62L**	ThermoFisher	12-0621-82	1:500
**FITC anti-CD25**	BioLegend	101907	1:500
**Pacific Blue anti-CD4**	ThermoFisher	MCD0428	1:500
**PE anti-CD8**	ThermoFisher	12-0081-85	1:500
**APC anti-IFN-γ**	ThermoFisher	17-7311-82	1:500
**APC-cy7 anti-CD4**	BioLegend	100526	1:500
**CellTrace TM CFSE Cell Proliferation Kit**	ThermoFisher	C34554	1:200
**Annexin V-FITC/PI**	MultiSciences	70-AP101-100	1:200
**PB anti-CD4**	BioLegend	100428	1:500
**APC-cy7 anti-CD8**	BioLegend	100713	1:500
**PE anti-human CD4**	ThermoFisher	12-0047-41	1:500

**FITC anti-human CD4**	ThermoFisher	11-0048-42	1:500
**APC anti-human Foxp3**	ThermoFisher	17-4777-42	1:500
**TGF-β1**	Cell Signaling	3711	1:1000
**TGF-β2**	Abclonal	A3640	1:1000
**TGF-β3**	Abclonal	A8460	1:1000
**β-Actin**	Cell Signaling	3700	1:1000
**p-Smad3**	Cell Signaling	9520	1:1000
**Smad3**	Cell Signaling	9523	1:1000
**p-PKA**	abcam	ab75991	1:3000
**PKA**	abcam	ab76238	1:3000
**LC3B**	Invitrogen	MA5-37852	1:3000
**ATG3**	Abclonal	A19594	1:1000
**ATG5**	Abclonal	A0203	1:1000
**K48-UB**	Cell Signaling	12805	1:1000
**K63-UB**	Cell Signaling	12930	1:1000
**UB**	Cell Signaling	3936	1:1000
**CHIP**	abcam	ab228742	1:3000
**Flag**	Cell Signaling	14793	1:1000
**His**	Cell Signaling	12698	1:1000
**Myc**	Cell Signaling	2276	1:1000
**HA**	Cell Signaling	3724	1:1000
**p-Ser/Thr**	Cell Signaling	9631	1:1000
**GM130**	Abclonal	A11408	1:1000
**Alix**	Abcam	ab275377	1:3000
**Tsg101**	Abcam	ab125011	1:3000
**CD63**	Abcam	ab217345	1:3000
**GAPDH**	Cell Signaling	5174	1:1000
**HRP-conjugated goat anti-mouse**	Cell Signaling	7076	1:1000
**HRP-conjugated goat anti-rabbit**	Cell Signaling	7074	1:1000

Supplementary Table 4: Information of sequences for real-time primers of PCR and sgRNA.

mRNA	Primers
***mActb* F**	5’-CGTTGACATCCGTAAAGACC-3’
***mActb* R**	5’-AACAGTCCGCCTAGAAGCAC-3’
***mFoxp3* F**	5’-CTCGTCTGAAGGCAGAGTCA-3’
***mFoxp3* R**	5’-TGGCAGAGAGGTATTGAGGG-3’
***mTgfb1* F**	5’-TGATACGCCTGAGTGGCTGTCT-3’
***mTgfb1* R**	5’-CACAAGAGCAGTGAGCGCTGAA-3’
***mTgfbr1* F**	5’-TGCTCCAAACCACAGAGTAGGC-3’
***mTgfbr1* R**	5’-CCCAGAACACTAAGCCCATTGC-3’
***mTgfbr2* F**	5’-CCTACTCTGTCTGTGGATGACC-3’
***mTgfbr2* R**	5’-GACATCCGTCTGCTTGAACGAC-3’
***mSmad7* F**	5’-GTCCAGATGCTGTACCTTCCTC-3’
***mSmad7* R**	5’-GCGAGTCTTCTCCTCCCAGTAT-3’
***mFos* F**	5’-GGGAATGGTGAAGACCGTGTCA-3’
***mFos* R**	5’-GCAGCCATCTTATTCCGTTCCC-3’
**#1 sgRNA for *Chip***	5’-AGTCAGCAAGTGCCTGTTCAGG-3’
**#2 sgRNA for *Chip***	5’-GGCAGTGTACTACACTAACCGG-3’
