# Supplementary material for: Ursodeoxycholic acid reduces antitumor immunosuppression by inducing CHIP-mediated TGF-β degradation
Source: Nat Commun. 2022 Jun 14;13:3419. doi: 10.1038/s41467-022-31141-6 (PMC9198048; doi:10.1038/s41467-022-31141-6)
Supplement: Supplementary file 3 — Description of Additional Supplementary Files [file 41467_2022_31141_MOESM3_ESM.docx]

**Description of Additional Supplementary Files**

**Supplementary Data 1**: Full MS analysis data in this study. MS analysis of proteins interacting with PKA in HEK293T cells in sheet 1; MS analysis of proteins interacting with TGF-β1 in HEK293T cells in sheet 2; MS analysis of immunoprecipitated TGF-β1 in HEK293T cells with TGF-β1 overexpression in sheet 3; MS analysis of immunoprecipitated TGF-β1 in HEK293T cells transfected with TGF-β1 and PRKACA overexpression in sheet 4.
